# Insect pest damage increases faba bean (*Vicia faba*) yield components but only in the absence of insect pollination

**DOI:** 10.1002/ece3.8686

**Published:** 2022-03-07

**Authors:** Laura G.A. Riggi, Chloé A. Raderschall, Ola Lundin

**Affiliations:** ^1^ 8095 Department of Ecology Swedish University of Agricultural Sciences Uppsala Sweden

**Keywords:** broad bean beetle, compensatory growth, ecosystem services, florivory, non‐additive effects, resource allocation

## Abstract

Identifying and quantifying crop stressors interactions in agroecosystems is necessary to guide sustainable crop management strategies. Over the last 50 years, faba bean cropping area has been declining, partly due to yield instabilities associated with uneven insect pollination and herbivory. Yet, the effect of interactions between pollinators and a key pest, the broad bean beetle *Bruchus rufimanus* (florivorous and seed predating herbivore) on faba bean yield has not been investigated. Using a factorial cage experiment in the field, we investigated how interactions between two hypothesized stressors, lack of insect pollination by bumblebees and herbivory by the broad bean beetle, affect faba bean yield. Lack of bumblebee pollination reduced bean weight per plant by 15%. Effects of the broad bean beetle differed between the individual plant and the plant‐stand level (i.e., when averaging individual plant level responses at the cage level), likely due to high variation in the level of herbivory among individual plants. At the individual plant level, herbivory increased several yield components but only in the absence of pollinators, possibly due to plant overcompensation and/or pollination by the broad bean beetle. At the plant‐stand level, we found no effect of the broad bean beetle on yield. However, there was a tendency for heavier individual bean weight with bumblebee pollination, but only in the absence of broad bean beetle herbivory, possibly due to a negative effect of the broad bean beetle on the proportion of legitimate flower visits by bumblebees. This is the first experimental evidence of interactive effects between bumblebees and the broad bean beetle on faba bean yield. Our preliminary findings of negative and indirect associations between the broad bean beetle and individual bean weight call for a better acknowledgment of these interactions in the field in order to understand drivers of crop yield variability in faba bean.

## Graphical abstract

Using a factorial cage experiment, interactions between pollinators and the key pest, Bruchus rufimanus, on faba bean yield were investigated. We find that faba bean yield can benefit from high levels of herbivory damage, but only in the absence of pollination. Furthermore, data shows a tendency for heavier beans with pollination when *B*. *rufimanus* was absent. These preliminary findings call for a better acknowledgment of these interactions in the field in order to understand drivers of crop yield variability in faba bean.

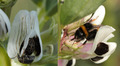

## 1 INTRODUCTION

Stressors are biotic or abiotic variables that cause a negative response in taxa or communities (Barrett et al., 1976; Vinebrooke et al., 2004). For pollinator‐dependent crops, insect herbivory and a lack of pollination can be referred to as biotic stressors if they negatively affect yield. Occasionally, herbivore‐attacked plants might yield more than un‐attacked plants (overcompensation, Poveda et al., 2010), and pollination benefits to yield might vary from negative to positive within and between cultivars (Bishop et al., 2020; Lundin & Raderschall, 2021). Therefore, the characterization of herbivory and lack of pollination as crop stressors is not clear cut, but rather a nuanced one that will depend on the frequency, timing, and quantity of herbivory and pollination, as well as modifiers such as nutrient availability and cultivar (Poveda et al., 2010). To characterize crop stressors, it is thus important to explore such nuances and investigate the interactions among multiple stressors that are acting simultaneously on crop yield (Peterson & Higley, 2000; Piggott et al., 2015). Empirically quantifying plant stressors and their interactions, particularly in agroecosystems, will help guide sustainable crop management strategies (Cote et al., 2016; Gagic et al., 2019; Saunders et al., 2016; Sutter & Albrecht, 2016).

Lack of insect pollination and insect herbivory may independently (additively), synergistically, or antagonistically affect yield of pollinator‐dependent crops, making net effects on crop yield challenging to predict (Figure S1). A synergistic effect between these stressors would result when the combined negative effect on yield, due to low insect pollination and high herbivory, is higher than the sum of their individual effects. This might result if herbivory negatively impacts insect pollination, for example, by reducing pollinator visitation rates to flowers (Moreira et al., 2019) or prompting pollinators to rob nectar instead of legitimately pollinating flowers (Ye et al., 2017). Alternatively, an antagonistic effect would result when yield loss due to lack of insect pollination and herbivory is lower than the sum of their individual effects. This might be the case if herbivore‐induced plant overcompensation has the capacity to minimize the negative effect of the lack of pollination (Järemo et al., 1999; Munguía‐Rosas et al., 2015) or if the herbivore directly benefits plant reproduction by acting as a pollinator (i.e., some florivores; see: McCall & Irwin, 2006). Interactions between insect pollination and herbivory have recently been found to influence plant traits (Ramos & Schiestl, 2019) and crop yield (Bartomeus et al., 2015; Gagic et al., 2019; Garibaldi et al., 2018; Lundin et al., 2013; Raderschall et al., 2021; Saunders et al., 2016; Sutter & Albrecht, 2016; Tamburini et al., 2019). Compensatory responses of crops to herbivory and effects of florivorous herbivores on yield are important and under‐investigated mechanisms as they can maintain or even increase yield of crops exposed to pests (Gagic et al., 2016; Poveda et al., 2018). A recent meta‐analysis found that overcompensation for insect herbivory in plants is pervasive and can increase crop yield (Garcia & Eubanks, 2019). For example, flower abortion due to herbivory can lead damaged plants to grow larger fruits (Sánchez & Lacasa, 2008) or produce more flowers (Peschiutta et al., 2020) than plants without herbivory. Despite yield increases, overcompensation due to herbivory may decrease yield quality (Peschiutta et al., 2020) and reduce the marketable crop. These herbivory effects in interaction with pollination can be particularly important in crops with large compensatory potential to biotic and abiotic stressors, such as faba bean (*Vicia faba* L.) (López‐Bellido et al., 2005).

Faba bean is an important nitrogen‐fixating legume crop grown worldwide (Jensen et al., 2010; Karkanis et al., 2018). Over the past 50 years, faba bean cropping area has been declining due to yield instabilities, associated to abiotic stress, pest and pathogen pressure (Karkanis et al., 2018), and possibly uneven insect pollination. Faba beans are partially dependent on insect pollinators (Bishop & Nakagawa, 2021), with honeybees and bumblebees being the dominant pollinators in Northern Europe (Nayak et al., 2015; Raderschall et al., 2021). While insect pollination generally increases faba bean yield and yield stability (Suso & Maalouf, 2010; Suso & del Río, 2015), pollination dependence within cultivars varies greatly, from −4 to 46% (loss in yield per plant without pollination) (Bishop et al., 2020). A recent study found that insect pollination benefit to faba bean, measured as the increase in bean weight per plant, lessened with aphid herbivory (Raderschall, Vico, et al., 2021). This variability in pollination benefit underlines the importance to investigate interactions between pollinators and major pests if we want to understand factors affecting yield variability in faba beans.

**FIGURE 1:**
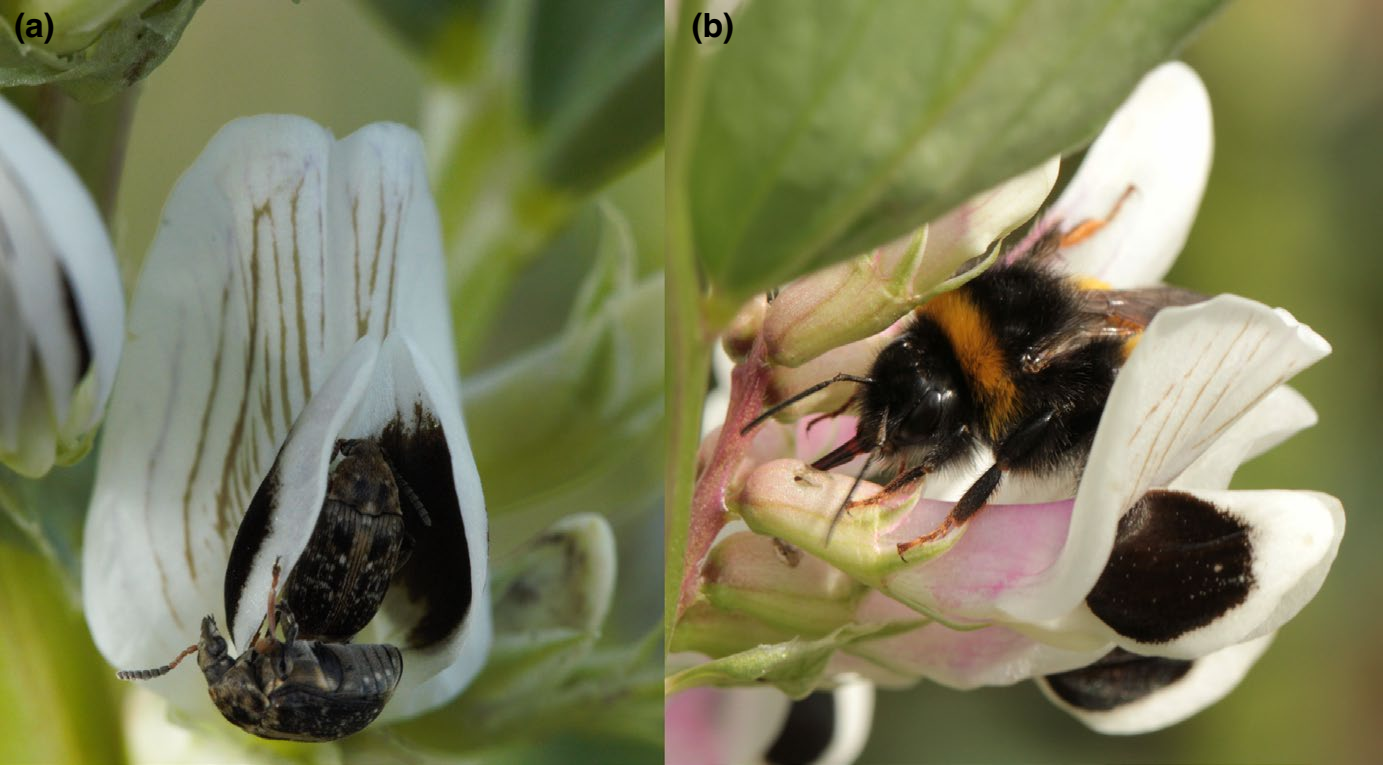
Organism photos of (a) *Bruchus rufimanus* in a faba bean flower and (b) *Bombus terrestris* robbing faba bean nectar from the base of the corolla (photos by C. A. Raderschall)

A key pest in faba bean is the broad bean beetle *Bruchus rufimanus* (Boh.) (Segers et al., 2021) (Figure 1). Adult beetles colonize the crop in spring to feed on pollen and nectar, and start laying eggs over a period of around 6 weeks (Segers et al., 2021). When the larvae hatch, they bore through the pods and develop and feed inside the beans. We use the term “herbivory” to include both florivory by the broad bean beetle adults and seed predation by the larvae. Larval feeding reduces seed weight and quality (Epperlein, 1992; Roubinet, 2016; Segers et al., 2021). Adult beetles might have additional negative effects on yield if their feeding on pollen disrupts pollinator visitation (Ye et al., 2017), or, alternatively, positive effects if they pollinate (Krupnick & Weis, 1999; McCall & Irwin, 2006). Interactions between pollinators and broad bean beetles on faba bean growth and yield have so far not been investigated.

Here, we evaluate the effect of herbivory by the broad bean beetle on faba bean yield components and investigate how interactions between two hypothesized stressors, namely lack of pollination and herbivory by the broad bean beetle, affect above‐ and belowground plant traits and yield of faba bean. Specifically, we investigate whether flower visitation by bumblebees changes with the inclusion of herbivores, and if there is over‐compensatory growth of the plant in response to broad bean beetles damage in the presence or absence of pollinators.

## 2 MATERIAL AND METHODS

### 2.1 Field experiment setup

To assess the individual and interactive effects of the two stressors, herbivory and lack of pollination on faba bean, we conducted a cage experiment in 2020 in a faba bean field in Uppsala, Sweden (59°50′29.12″N; 17°42′02.44″E). We conducted a fully‐crossed two‐factor field experiment, with presence and absence of herbivores (H+/H−) and pollinators (P+/P−), where H+ and P− were the stressed level of each factor. Each H and P treatment combination had seven replicates (N = 28 in total), arranged in seven blocks, with one treatment combination per block (Figure S2). We used 2 × 2 × 2 m cages covered with a nylon net (mesh size: 0.6 × 0.6 mm) to control the access of the herbivores and pollinators to the crop. Faba bean seeds (cultivar: Tiffany, Scandinavian Seed; pollinator dependency: 61% increase in bean mass per plant (Raderschall, Vico, et al., 2021)) were planted in the field on the 24th of April (plant density per m^2^: mean ± SD = 58.4 ± 8.5), and plants were treated with the fungicide Signum (0.5 kg ha^−1^, BASF; 267 g kg^−1^ boscalid +67 g kg^−1^ pyraclostrobin) on the 15th of June, before flowering (BBCH‐51) and prior to bumblebee hive inclusion, to avoid negative impacts on pollinators (Fisher et al., 2021). The experiment was conducted in a field where no faba bean had been planted in the previous year to avoid overwintering broad bean beetle emergence in the cages.

### 2.2 Herbivore inoculation

Broad bean beetles were collected over 2 weeks in early June from faba bean crops in the southern region of Västergötland, where they had already colonized the earlier flowering fields. In the H+ treatment cages (N = 14), 45 broad bean beetle individuals per m^2^ (0.77 individuals per plant) were inoculated on the 16th of June, before crop bloom (BBCH‐51:59). This pest density was chosen based on surveys of broad bean beetles conducted by the Swedish Board of Agriculture in 55 faba bean fields across Sweden between 2016 and 2019, which found that maximum naturally occurring pest incidences in commercial faba bean fields were 0.5–1 broad bean beetle per plant, depending on the region. Broad bean beetles were in the cages until the end of the experiment (for a duration of 3 months); mating and oviposition span 2 and 6 weeks, respectively (Segers et al., 2021). After oviposition, adults might migrate to feed on flowers nearby the crop before dying (Segers et al., 2021); however, in the cages adults were limited to feed on the extra pollen given to the bumblebees. the percent of bean damage caused by oviposition in the cages was comparable to damage levels found during the field surveys conducted by the Swedish Board of Agriculture. To control for herbivores in the H‐ treatment (N = 14), cages were checked for broad bean beetles and any individual was removed prior to pollinator supplementation. No other faba bean crop pests, such as aphids, were detected in the cages.

### 2.3 Pollinator supplementation and flower visitation

To create a P+/P− treatment, we supplemented P+ cages (N = 14) with bumblebee hives (*Bombus terrestris* L., Natupol Seed, Koppert, https://www.koppert.com/natupol‐seeds/) on the 22nd of June at the onset of flowering (BBCH‐61). *Bombus terrestris* was chosen as a model species as it is commercially available, benefits faba bean yield (Bishop et al., 2016; St‐Martin & Bommarco, 2016), and is one of the most common flower visitors of faba bean in Scandinavia (Lundin & Raderschall, 2021). The hives contained approximately 2–5 workers foraging for pollen and nectar and 5–8 males collecting nectar, simulating a high pollinator abundance scenario. Pollinators were supplemented with sugar water and pollen to minimize over visitation. Hives were placed at 1 m above the ground facing east for the duration of crop flowering, until the 27th of July. At the end of the experiment, we found high variation in the abundance of bumblebees inside each hive (mean ± SD = 13.6 ± 5.1), but there were no differences in bumblebee abundances between H+ and H− treatments (*t*‐test: *p *= .4), and bumblebee abundances in the hives were not correlated with pollinator visitation rates (Pearson rho: −0.14).

To investigate the effects of broad bean beetles on bumblebee pollinators, we carried out pollinator visitation observations in each P+ cage in both herbivory treatments (N = 14). Between the 23rd of June and the 10th of July, pollinators in P+ cages were surveyed 15 times. Surveys were carried out under good weather conditions (>15°C and no rain) between 1 and 6 p.m. After each survey, the number of open flowers were counted, initially on 10 plants and from the 7th of July, when flowering was decreasing (survey round 7), in a 1 m^2^ quadrat (¼ of the cage). The same plants and quadrat were observed in every survey round. Pollinator visitation rate per flower and foraging behavior were recorded for a duration of 10 min, initially on the 10 plants, where the number of flowers had been counted and later in the 1 m^2^ quadrat. For each visit, we noted whether pollinators were legitimately visiting flowers by inserting their proboscis through the front of the flower opening, visiting extra floral nectaries (EFN) located underneath the stipules, or robbing nectar by inserting their proboscis through a hole at the base of the flower tube (Tasei, 1976). Because of low sugar‐water reserves in all the hives due to spillage during transportation, pollinator behavior might have been affected in the first half of the experiment, with more nectar robbing behavior than would have been the case if bumblebees had not been sugar‐starved. From the 7th of July, each colony was supplemented with sugar‐water. The timing of sugar‐water supplementation was included as a factor in the analyses of pollinator visitation and foraging behavior.

### 2.4 Plant measurements

We estimated plant density by counting the number of plants within a 0.25 m^2^ quadrat randomly placed in each cage. When pods reached maturity (BBCH‐89), on 10th of September, 20 plants (stem, leaves, pods and roots) were collected per cage. On each plant, we counted the number of pods, number of beans per pod, proportion of damaged beans (beans with broad bean beetle emergence holes), plant height, and tap root length. Pods were classified into three categories: mature, immature (small and green), and unfertilized (without beans inside). Roots were washed with water. Aboveground plant (stems and leaves) and root biomass and bean weight for each plant were dried at 65°C for 48 h and subsequently weighed. Beans per pod was averaged per plant prior to statistical analyses, beans per plant was calculated by summing the number of beans across pods per plant, and mean individual bean weight was calculated by dividing bean weight per plant with number of beans per plant. Yield in dt ha^−1^ was calculated for each cage by multiplying average bean weight per plant with plant density.

### 2.5 Data analyses

We used generalized linear mixed‐effects models to test the interactive effects of herbivory and pollination treatments on: (a) proportion of damaged beans per plant (beans with broad bean beetle emergence holes), (b) faba bean yield components (individual bean weight, total bean weight per plant, number of beans per pod, number of beans per plant, number of pods (mature, immature and unfertilized) per plant, proportion of mature pods per plant, and yield (dt ha^−1^)), and (c) plant growth components (plant height and aboveground biomass, root length and biomass). All variables follow normal distributions except for number of pods and beans per plant, where a Poisson distribution was assumed or a negative binomial distribution when data was overdispersed, and for proportion of damaged beans and mature pods per plant where a binomial distribution was assumed (see Table 1 for model structures). The explanatory variables in all models included the H+/H− and P+/P− treatments and their interaction term. Despite the care taken to remove broad bean beetles from the H‐ cages at the beginning of the experiment, beans with emergence holes were also found in these cages, and there was a large variation in damage between plants within each H+/H− cage (Figure S3). In particular, cage 3, which belongs to the P+H+ combination, had a low proportion of damaged beans, almost comparable to the level of damage observed in the H− combinations. We therefore analyzed the data at the plant‐stand level with and without cage 3 and provided results for qualitative differences between these analyses. In addition to the main H+/H− treatment effect (i.e., plant‐stand level, measured by averaging individual plant level responses in each cage), we also investigated the effect of herbivory damage at the plant level, measured as percentage of damaged beans per plant within cage (% Damage). Proportion of damaged beans did not vary with pollination treatment levels (Table 1, *p *= .09). We tested all plant yield and growth variables, and used P+/P− treatment and % Damage per plant and their interaction term as explanatory variables. The random structure in all models included cage identity (N = 28) nested within block (N = 7), except for yield (dt ha^−1^) per cage where only block was included. If significant interactions were found, post hoc tests using the “emmeans” package were carried out to investigate the direction of the effect.

**TABLE 1:** Model outputs for both plant‐stand (cage) and plant level analyses

	Variables		Stand level					Plant level			
			H+	P+	H*P	*R*_m_^2^		%Damage	P+	%Damage*P	*R*_m_^2^
Bean 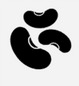	***Proportion of damaged beans^c^	Est ± SE	3.85 ± 0.64	−1.17 ± 0.70	0.98 ± 0.93	0.59					
		*p*	**<.01**	.09	.29						
	Individual bean weight (g)^a^ *	Est ± SE	0.01 ± 0.02	0.06 ± 0.02	−0.06 ± 0.03	0.03		0.001 ± 0.0004	0.07 ± 0.01	−0.002 ± 0.0006	0.04
		*p*	.59	.**01**	.06			**<.01**	**<.01**	**<.01**	
	Bean weight per plant (g)^a^	Est ± SE	−0.26 ± 1.28	3.01 ± 1.28	−1.74 ± 1.81	0.03		0.05 ± 0.01	3.57 ± 1.13	−0.06 ± 0.02	0.04
		*p*	.83	.**03**	.34			**<.01**	**<.01**	.**02**	
	Beans per pod^a^	Est ± SE	0.1 ± 0.14	0.57 ± 0.14	−0.2 ± 0.2	0.07		0.009 ± 0.002	0.65 ± 0.12	−0.008 ± 0.003	0.11
		*p*	.48	**<.01**	.31			**<.01**	**<.01**	**<.01**	
	Beans per plant^b^	Est ± SE	−0.009 ± 0.1	0.21 ± 0.1	−0.03 ± 0.14	0.03		0.004 ± 0.001	0.29 ± 0.09	−0.004 ± 0.002	0.05
		*p*	.92	.**03**	.83			.**01**	**<.01**	.10	
	Yield (dt/ha)^a^	Est ± SE	−4.13 ± 6.79	11.96 ± 6.79	−7.50 ± 9.60	0.2					
		*p*	.54	.09	.44						
Pods 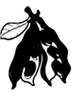	Proportion of mature pods ^c^	Est ± SE	−0.23 ± 0.55	1.71 ± 0.57	0.07 ± 0.81	0.19		0.07 ± 0.005	2.00 ± 0.61	−0.008 ± 0.008	0.54
		*p*	.67	**<.01**	.92			**<.01**	**<.01**	.33	
	Pods per plant^b^	Est ± SE	−0.02 ± 0.06	−0.02 ± 0.06	0.01 ± 0.09	<0.01		−0.001 ± 0.001	−0.01 ± 0.07	−0.00007 ± 0.002	0.01
		*p*	.67	.66	.85			.13	.79	.96	
	Mature pods per plant^b^	Est ± SE	−0.07 ± 0.11	0.16 ± 0.1	0.04 ± 0.15	0.03		0.007 ± 0.002	0.36 ± 0.11	−0.007 ± 0.003	0.07
		*p*	.49	.12	.78			**<.01**	**<.01**	**<.01**	
	Immature pods per plant^d^	Est ± SE	0.51 ± 0.54	−2.07 ± 0.66	0.26 ± 0.89	0.34		−0.06 ± 0.007	−1.89 ± 0.72	−0.02 ± 0.08	0.55
		*p*	.34	**<.01**	.76			**<.01**	**<.01**	.12	
	Unfertilized pods per plant^d^	Est ± SE	−0.14 ± 0.6	−1.35 ± 0.61	0.28 ± 0.87	0.14		−0.03 ± 0.003	−0.95 ± 0.52	−0.01 ± 0.008	0.62
		*p*	.80	.**02**	.74			**<.01**	.07	.057	
Plant 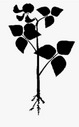	Plant biomass (g)^a^	Est ± SE	0.15 ± 0.29	−0.99 ± 0.29	−0.41 ± 0.42	0.06		−0.01 ± 0.005	−1.32 ± 0.28	0.005 ± 0.008	0.08
		*p*	.61	**<.01**	.34			.**01**	**<.01**	.48	
	Plant height (cm)^a^	Est ± SE	0.46 ± 2.18	−9.1 ± 2.18	−1.77 ± 3.09	0.17		−0.14 ± 0.02	−11.89 ± 1.98	0.09 ± 0.04	0.25
		*p*	.83	**<.01**	.57			**<.01**	**<.01**	.052	
	Root biomass (g)^a^	Est ± SE	−0.14 ± 0.15	−0.20 ± 0.15	−0.03 ± 0.21	0.01		−0.003 ± 0.003	−0.21 ± 0.14	−0.0004 ± 0.004	0.02
		*p*	.36	.19	.88			.29	.14	.92	
	Root length (cm)^a*^	Est ± SE	−1.29 ± 0.66	0.11 ± 0.63	0.61 ± 0.91	0.01		−0.02 ± 0.01	0.57 ± 0.60	−0.008 ± 0.01	0.03
		*p*	.06	.85	.51			.**02**	.35	.62	

Note

Presented are the yield and plant growth components with respect to pollination treatment (P+) and herbivory treatment (H+) or herbivory damage (% of beans with broad bean beetle emergence holes per plant, % Damage) and their two‐way interactions. Shown are mean estimates (Est) for the respective treatments, standard errors (SE), *p*‐values (*p*), and the adjusted marginal *R* ^2^ (*R*_m_^2^) of the model. Significant results (*p *< .05) are in bold. Plots for significant results and raw data are shown in Figures S4–S5.

^a^ Linear mixed model with normal distribution (“lme”).

^b^ Generalized linear mixed model (glmm) with a negative binomial distribution (“glmer.nb”). Units on log scale.

^c^ Glmm with binomial distribution (“glmer”). Units on log‐odds scale.

^d^ Glmm with Poisson distribution (“glmer”). Units on log scale.

* Results differ qualitatively for plant‐stand level analyses after removal of cage 3 (Table S1).

To test the effects of herbivory on observed pollinator behavior (proportion of legitimate, robbing, and EFN visits) and on pollinator visitation rate (legitimate visits per flower per time unit), we used a generalized mixed‐effects model with a binomial and a normal distribution, respectively. The explanatory variables included H+/H− treatment and the number of open flowers per m^2^ and their interaction term. To account for addition of sugar‐water to the pollinators on the 7th of July, a binary factor (sugar‐water: yes/no) was included as well as its interaction with number of open flowers per m^2^ and herbivory treatment. The interaction between herbivory treatment and sugar‐water was never significant and did not improve the models as determined by the Akaike Information Criterion (AIC), indicating that the effect of herbivory on pollinator behavior did not change after the addition of the sugar‐water. This interaction was therefore excluded. To investigate the effect of herbivores on number of open flowers per m^2^ in cages with pollinators, we used a generalized mixed‐effects model with H+/H− treatment as explanatory variable. The random structure for all models incorporated the sampling round (N = 15) nested within cage identity and block.

The residuals of all models were visually inspected to validate the model assumptions and, additionally, generalized linear models were checked for overdispersion using “DHARMa” (Hartig & Lohse, 2020). Multicollinearity was checked for all models (variation inflation factor <2). All analyses were conducted in R version 3.6.3, using packages “nlme” (Pinheiro & Bates, 2020), “lme4” (Bates et al., 2020), “emmeans” (Lenth et al., 2021), and “ggplot2” (Wickham et al., 2020) to plot data.

## 3 RESULTS

### 3.1 Yield and its components

**FIGURE 2:**
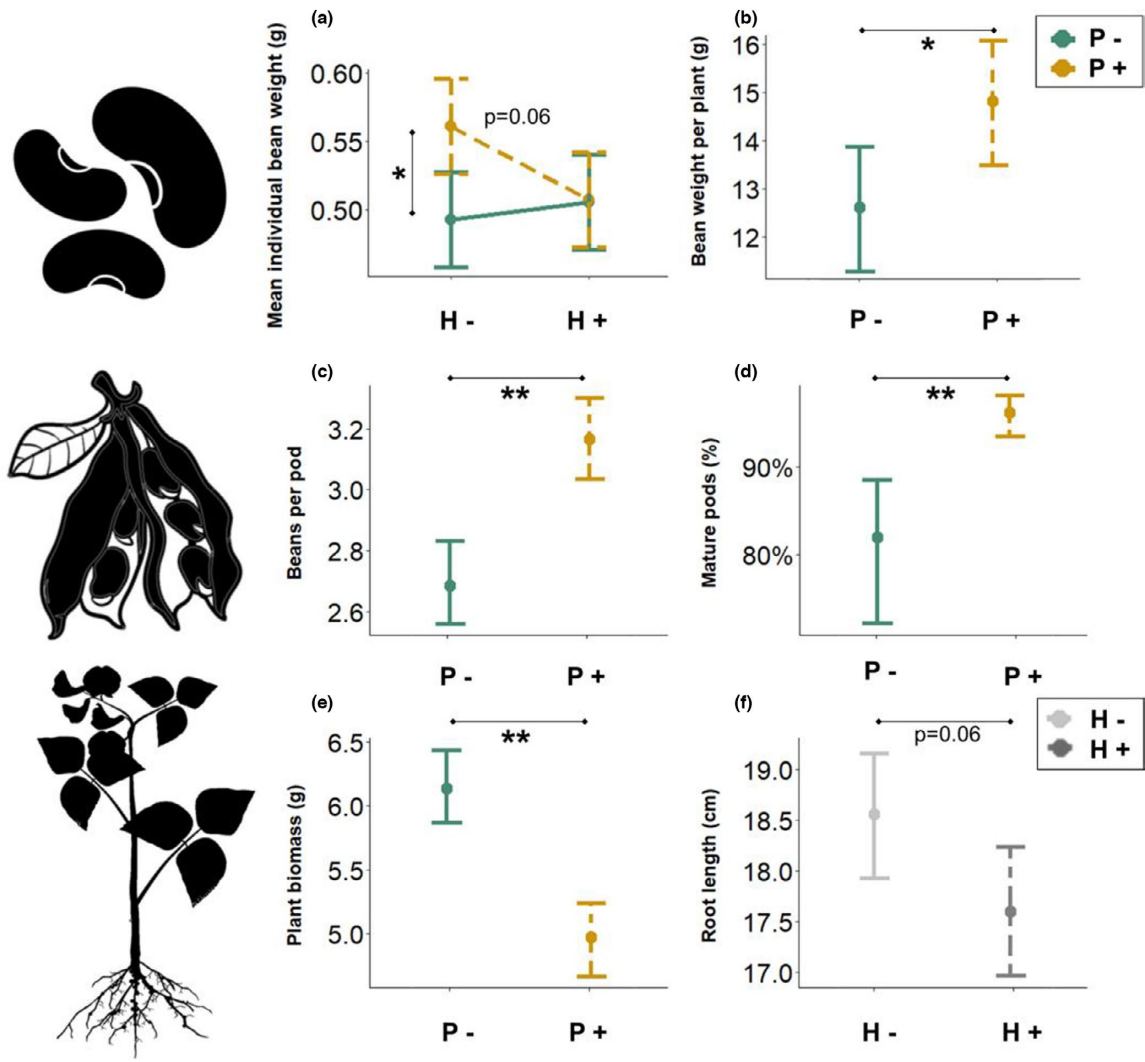
Model prediction for faba bean yield and growth components in relation to herbivory (**H−**: solid light gray line; and **H+**: dashed dark gray line) and pollination (**P−**: solid green line; and **P+**: dashed yellow line) levels: (a) mean individual bean weight (g), (b) bean weight per plant (g), (c) number of beans per pod, (d) percentage of mature pods per plant, (e) aboveground plant dry biomass (g), and (f) tap root length (cm) per plant. Whiskers represent 95% confidence intervals. Significance codes: “*” < .05; “**” < .01 (Table 1). Note that y‐axes do not start at zero

Lack of pollination (P−) decreased total bean weight per plant, mean number of beans per pod, and total number of beans per plant, independently of the herbivory treatment (Table 1, Figures 2b,c, S4). In the absence of insect pollination, bean weight and number of beans per plant decreased by 15% and 17%, respectively. There was a marginal (*p *= .06) interactive effect of pollination and herbivory on individual bean weight, with pollination increasing individual bean weight but only in the absence of herbivory (Table 1, Figures 2a, S4). This interaction was significant after removal of cage 3 (est = −0.07, SE = 0.03, *p* = .03), which belonged to the P+H+ combination but had low proportion of damaged beans (Figure S3, Table S1). Herbivory increased the percentage of damaged beans, from 3% in the H− treatment to 40% in the H+ treatment (Table 1, Figure S3), which is over tenfold the economic injury threshold set for beans targeting human consumption and could lead to up to 78% economical losses (Bachmann et al., 2020; Roubinet, 2016). There was neither an effect of pollination nor of herbivory treatments on yield (dt ha^−1^), or mature or total number of pods per plant; however, the proportion of mature pods was lower with lack of pollination, due to higher numbers of unfertilized and immature pods (Table 1, Figures 2d, S4).

**FIGURE 3:**
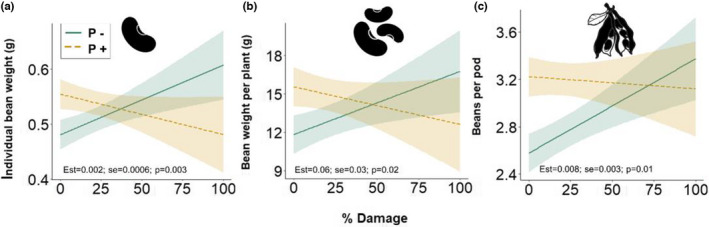
Model predictions for faba bean yield components in relation to herbivory damage (% of beans with broad bean beetle emergence holes per plant) and pollination levels (**P−**: solid green line; and **P+**: dashed yellow line): (a) individual bean weight (g) (significant differences between the pollination treatments for Damage levels <18% and >83% (“emmeans”)), (b) bean weight per plant (g) (significant differences between the pollination treatments for Damage levels <23%) and (c) beans per pod (significant differences between the pollination treatments for Damage levels <44%). Est ± SE and *p*‐values for the differences of the simple slopes (“emtrends”) are presented in each panel. Bands represent 95% confidence intervals. Note that y‐axes do not start at zero

When herbivory damage on each plant was used as an explanatory variable (% Damage), there were interactive effects of pollination and herbivory damage on individual bean weight, total bean weight per plant, and number of beans per pod (Table 1, Figures 3, S5). In the absence of pollination, there were positive relationships between yield and herbivory damage for several yield components (individual bean weight: est = 0.001, SE = 0.0004, *p *< .01; total bean weight: est = 0.06, SE = 0.02, *p *< .01; and number of beans per pod: est = 0.007, SE = 0.002, *p *< .01) (Figures 3, S5). In the presence of insect pollination, there were no relationships with herbivory damage (Figures 3, S5). In addition, number of beans per plant increased with increasing herbivory damage independent of pollination treatment (Table 1, Figure S5). Number of pods per plant was not affected, but proportion of mature pods increased with herbivory damage due to a decrease in the number of unfertilized and immature pods (Table 1).

### 3.2 Plant growth

Plant aboveground biomass and height were higher with lack of pollination (Table 1, Figures 2e, S4). While there was no effect of pollination or herbivory on root biomass, root length was marginally (*p *= .06) shorter in the presence of herbivory (Table 1, Figures 2f, S4); this effect became significant when considering broad bean beetle% Damage as an explanatory variable (Table 1, Figure S5). In addition, there was a negative relationship between herbivory damage and plant aboveground biomass and height (Table 1, Figure S5). The effect of herbivory on root length was significantly negative after removal of cage 3 (est = −1.29, SE = 0.65, *p* = .048), which belonged to the P+H+ combination but had low proportion of damaged beans (Figure S3, Table S1).

### 3.3 Flower visitation

**FIGURE 4:**
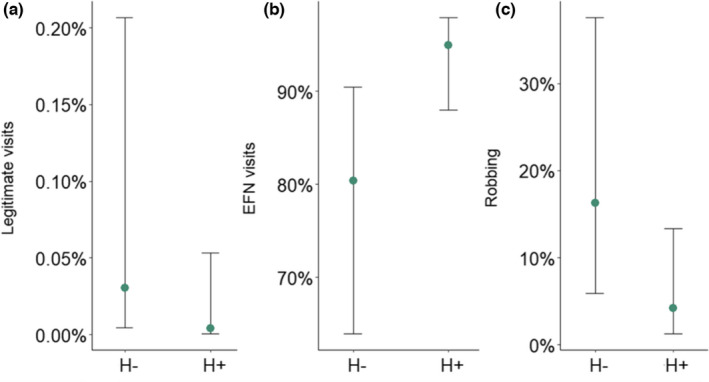
Model prediction for percent of (a) legitimate flower visits, (b) EFN visits, and (c) robbing by bumblebees in relation to a. herbivory (H−/H+) levels. Whiskers represent 95% confidence intervals. Note that the y‐axis in panel b. do not start at zero

Addition of sugar‐water increased flower visitation rate (est = 0.36, SE = 0.11, *p *= .01) and the proportion of legitimate visits (est = 10.4, SE = 2.6, *p *< .01) as expected. Despite a low flower visitation rate (mean = 0.03, SD = 0.08, min = 0, max = 0.66 visits per flower per 10 min) and no effect of herbivory on visitation rate (est = −0.05, SE = 0.04, *p* = .31), there was a negative effect of herbivory on proportion of legitimate flower visits by bumblebees (mean ± SE = 0.27 ± 0.18%, est = −2.03, SE = 0.89, *p* = .022, *R* ^2^_m_ = 0.05), and a positive effect on proportion of EFN visits (mean ± SE = 84 ± 3.7%, est = 1.34, SE = 0.53, *p *= .011, *R* ^2^_m_ = 0.11) but not robbing (mean ± SE = 3.2 ± 1.4%, est = −1.47, SE = 0.81, *p* = .07) (Figure 4). While nectar robbing increased with the number of open flowers per m^2^ (est = 0.02, SE = 0.01, *p* = .03, Figure S6), visitation rate and proportion of legitimate visitation were negatively affected by the number of open flowers per m^2^ but primarily after sugar‐water was added (interaction term: visitation rate est = −0.004, SE = 0.002, *p* = .073, *R* ^2^_m_ = 0.10; proportion of legitimate visits est = −0.09, SE = 0.04, *p* = .039, *R* ^2^_m_ = 0.42) (Figure S6). However, there was no effect of herbivory on the number of open flowers per m^2^ (est = −4.69, SE = 5.55, *p *= .43, *R* ^2^_m _= 0.01).

## 4 DISCUSSION

**FIGURE 5:**
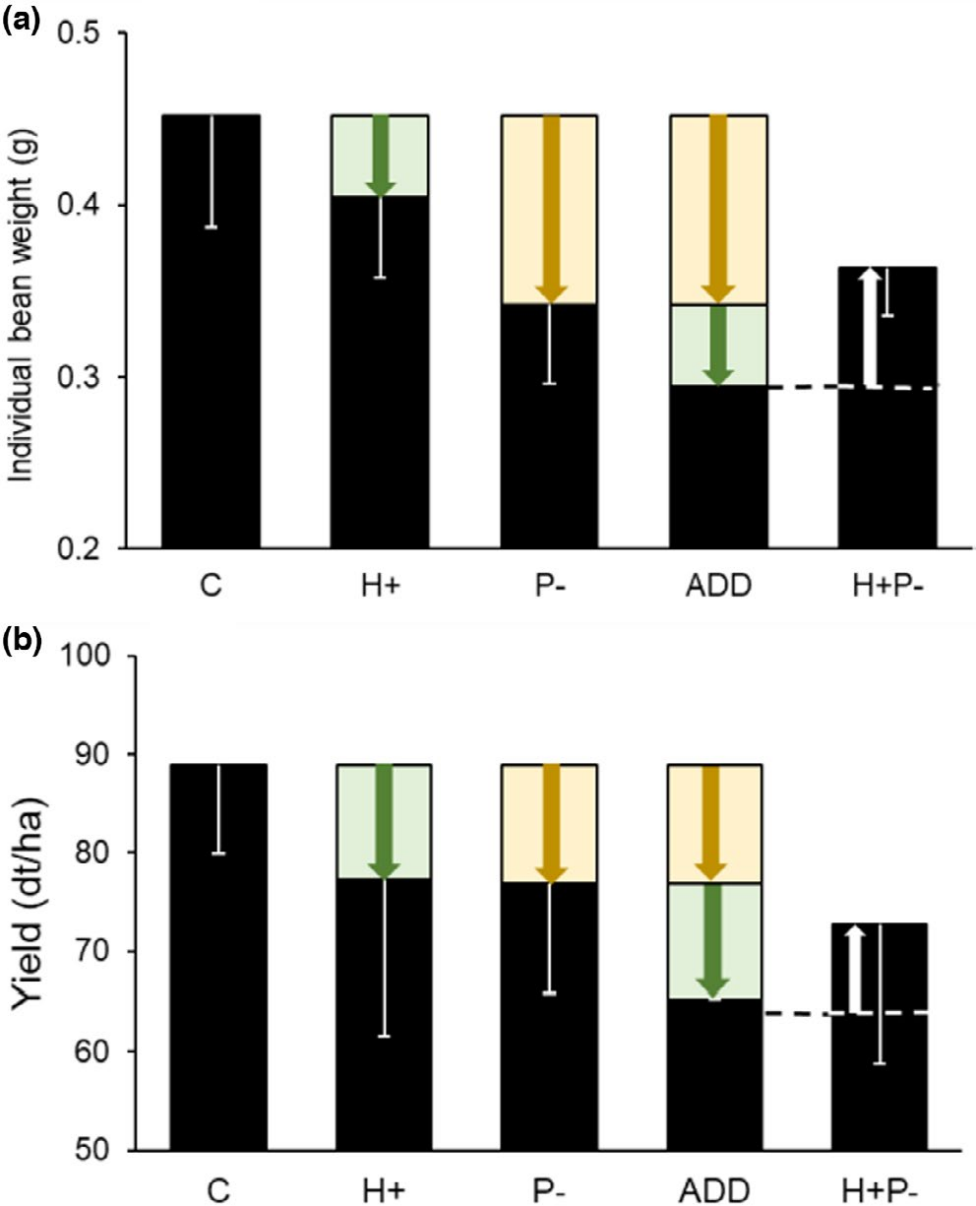
Diagram of interaction of stressors on (a) individual bean weight (g) and (b) crop yield (dt/ha) using raw data for each treatment (i.e., H+P+, H+P−, H−P+, H−P−). Treatments are non‐stressed control (C = H−P+) and stressors herbivory (**H+**P+) and lack of pollination (H−**P−**). An interaction is additive (ADD) if response to the application of both stressors is the sum of the effects of both treatments (Figure S1). The dashed line shows this additive prediction. As the observed change in individual bean weight (a) and crop yield (b) when both stressors are combined (**H+P−**) is less than the sum of the effects of both stressors, the response was numerically antagonistic, but the interaction was not statistically significant for yield (Table 1). Error bars indicate standard deviations

We investigated how interactions between two hypothesized faba bean stressors—herbivory and lack of insect pollination—affect yield components (Figure 5). While lack of insect pollination is a clear stressor of faba bean, as it related negatively to several yield components, effects of herbivory by the broad bean beetle were less straightforward despite high levels of damage. Effects of the broad bean beetle differed between the individual plant and plant‐stand level, when averaging individual plant level responses at the cage level. No synergies were observed between the two stressors, lack of insect pollination and herbivory. At the plant‐stand level, effects between stressors were mostly additive, except for individual bean weight where antagonistic effects were observed (Figure 5). At the plant level, interactions were mostly antagonistic, with increasing broad bean beetle damage increasing yield components but only in the absence of pollinators (Figure 3). No negative effects of the broad bean beetle on yield components were found at the plant‐stand level. Differences between the plant‐stand and plant level analyses in the effect of the broad bean beetle are likely due to high variation in the level of herbivory damage among individual plants within cages (Figure S3).

Despite high variation between faba bean cultivars in their dependency on pollinators (Bishop et al., 2020; Bishop & Nakagawa, 2021), lack of insect pollination generally leads to lower yield in faba bean crops (Raderschall, Vico, et al., 2021; Suso et al., 1996; Suso & del Río, 2015). This study confirms that lack of pollination decreases several faba bean yield components and is in line with a recent cage experiment using the same cultivar (Tiffany), where bean weight per plant and beans per pod but not individual bean weight benefitted from insect pollination (Raderschall, Vico, et al., 2021). In the absence of pollinators, the bean weight and the number of beans per plant decreased by 15% and 17%, respectively. Costs of lacking insect pollination were evident despite high frequencies of *B*. *terrestris* robbing, a common behavior in this crop (Marzinzig et al., 2018), indicating that legitimate flower visits were sufficient despite high robbing, or supporting the idea that robbing may benefit yield by increasing self‐pollination in faba bean, possibly by shaking pollen from the anthers on to the stigma (Kendall & Smith, 1975). Counterintuitively, visitation rate and proportion of legitimate flower visits decreased with number of open flowers (after sugar‐water addition), while proportion of nectar robbing increased (Figure S6). This is likely due to nectar deprivation at the start of the study which meant that bumblebees shifted from robbing nectar to legitimate visit later in the season when there were fewer flowers open. We found a modest decrease in bean weight per plant due to lack of pollination (15%) compared to results from an experiment using the same cultivar and bumblebee hives (61%, Raderschall, Vico, et al., 2021) or a recent meta‐analysis on faba bean pollinator dependency (37% on average, Bishop & Nakagawa, 2021). In addition, a recent field study using the cultivar Tiffany found no dependence on insect pollination (Lundin & Raderschall, 2021), indicating high variation in pollination benefit within this cultivar, likely mediated by environmental conditions. Finally, despite observed yield benefits with *B*. *terrestris* in this experiment, field studies have found that less frequent and more specialized pollinator species with long tongues, such as *B*. *hortorum*, improve pollination and cross‐fertilization of faba bean compared to *B*. *terrestris* (Marzinzig et al., 2018). Further studies investigating pollination and herbivory interaction in faba bean should include a greater diversity of pollinator species varying in their behavior.

Despite high levels of damage, there were no negative effects of the pest *B*. *rufimanus* on crop weight. While there was a higher proportion of damaged beans in the presence of broad bean beetles, which meant that the plant stand was non‐marketable for human consumption due to aesthetic damage to the beans (>3% damage, beans are then used for animal fodder and achieve a much lower price, Bachmann et al., 2020; Roubinet, 2016) (Figure S3b), this did not translate to a lower total or individual bean weight, number of beans or pods per plant. This result contrasts the findings of a recent study considering aphid herbivory, and which shows that bean aphid herbivory reduced all yield components (Raderschall, Vico, et al., 2021). An economic analysis would be valuable to compare faba bean yield losses due to aphids and yield value losses due to broad bean beetle damage. However, we found a marginal interaction (*p *= .06), which was significant after exclusion of cage 3 from the analysis, between pollination and herbivory on individual bean weight, whereby individual bean weight was heavier with pollination, but only when herbivory was absent. While we found no evidence that bumblebees legitimately visited flowers less frequently, proportion of legitimate flower visits decreased and EFN visits increased in the presence of broad bean beetles. This change in behavior might be a consequence of direct interference, lower pollen availability, and/or morphological alteration of the flower caused by the broad bean beetle feeding. We did not observe any flower damage in H+ cages (pers. obs.), indicating that direct interference or pollen depletion play a more important role. There is, to our knowledge, no literature investigating damage to flowers or pollen depletion by broad bean beetle adults. Insect pollination has been shown to increase individual bean weight (Suso & del Río, 2015); therefore, reduced proportion of legitimate flower visits due to broad bean beetles could lead to lower individual bean weight. This interaction was, however, not apparent on other yield components, such as the number of beans per pod and per plant. This might be because the number of beans is primarily determined by ovule fertilization (pollination), while individual bean weight might also be affected by plant resource allocation after fertilization, which could have been adversely impacted by the presence of the broad bean beetle (Patrick & Stoddard, 2010). Adverse herbivory effects of leaf chewers and sap feeders on bean weight have been found in *Fabaceae* (Cuny et al., 2018; Raderschall, Vico, et al., 2021). Therefore, resource allocation to developing beans might have been compromised by the broad bean beetle and lead to lower individual bean weight when pollinators were present.

We detected a high variation in broad bean beetle damage between plants within cages (Figure S3). When analyzing the effect of the broad bean beetle, at the individual plant level we detected interactions and, generally, positive relationships between herbivory damage and yield components, but only in the absence of bumblebee pollination. This indicates that the level of damage caused by the broad bean beetle affects plant resource allocation, and that this response depends on the presence of pollinators. Two non‐exclusive mechanisms, plant overcompensation to herbivory (i.e., damaged plants have higher fitness than undamaged plants) and pollination by broad bean beetle adults, explaining these results are discussed below.

A biological process, which potentially underlies the differential response to herbivory depending on pollination treatment, is the capacity of faba bean plants to overcompensate for damage or stress (López‐Bellido et al., 2005). Faba bean might overcompensate for early and high pod damage by the broad bean beetle by increasing pod production or bean weight during their growth. This is because the growth of faba beans is indeterminate, and while pods are developing at the lower nodes, flower production continues at the upper ones. This leads to competition for resources within the plant between the first set of pods, the roots, bean growth, as well as with the developing pods further up the stem (Jaquiery & Keller, 1978; Smith, 1982). Roots were on average 5% shorter in the presence of broad bean beetles and root length correlated negatively with herbivory damage. This indicates alterations in resource allocation from roots to other plant parts in the presence of broad bean beetles (Heinze, 2020). Further studies quantifying faba bean root nodules in interaction with herbivory and lack of pollination would shed some light on plant resource acquisition and allocation in relation to biotic stress. Plants might have overcompensated for herbivory damage in the absence of pollinators, as several yield components in highly damaged plants were higher compared to undamaged plants (Figure 3). Overcompensation in terms of seed‐set in response to herbivory is common in other crops such as *Brassicaceae* (Gagic et al., 2016; Rusman et al., 2018). Increase in yield components with increasing broad bean beetle damage was only visible in the absence of pollinators, when plants were stressed by both a lack of pollination and high herbivory damage. We found that in the absence of pollinators, faba bean plants had a higher aboveground biomass, likely to compensate for insufficient pollination by increasing the production of new pods at the upper nodes (Raderschall, Vico, et al., 2021). In addition, we found higher numbers of immature/unfertilized pods in the absence of pollinators, indicating that in the absence of pollinators the plants were still producing new pods at the time of harvest. Prolonged pod production in the absence of pollinators (i.e., after the broad bean beetle oviposition period) and plant compensation to herbivory through increased pod production or bean weight might explain increases in yield components with increasing herbivory but only in the absence of pollinators. Another hypothesis is that higher plant biomass, in the absence of pollinators, might enhance the plant's photosynthetic capacity and increase its ability to overcompensate to *B*. *rufimanus* damage. Indeed, plants can increase photosynthetic activity to recover fitness from herbivory damage (Stowe et al., 2003). However, very little is known about how plants optimize their resource allocation under multiple stressors.

Another non‐exclusive biotic process that might explain the observed increases in yield with increasing herbivory in the absence of bumblebee pollination is pollination by the broad bean beetle. Indeed, florivorous herbivores potentially act as pollinators if they transfer pollen between (cross‐pollination) or within (self‐pollination) flowers. For example, damage by the bud‐clipping weevil *Anthonomus signatus* lead to an increase in self‐pollination in strawberries (Penet & Collin, 2009), and adult pollen beetle, *Brassicogethes aeneus*, feeding has also been shown to pollinate oilseed rape (Williams, 2010). Faba bean plants that had greater level of herbivory damage might have had flowers that were visited more often by broad bean beetle adults, leading to positive associations between damage and pollination by the broad bean beetle and increased yield components in highly damaged plants. The positive effects of the broad bean beetle on yield were only visible in the absence of bumblebee pollination, an indication that when more efficient pollinators are present, broad bean beetles do not benefit faba bean pollination. While the net effect of bumblebees on yield components was positive in the presence of both bumblebees and broad bean beetles, some plants likely received high numbers of visits, due to the supplied high bumblebee densities, and sustained more flower damage than what they could compensate for (Sáez et al., 2014), leading to lower bean weight per plant in the pollination treatment under high levels of herbivory damage. The fact that bumblebees mainly robbed nectar instead of conducting legitimate visits could have influenced the results. Indeed, we hypothesize that more legitimate visits would lead to higher rates of cross‐pollination and subsequent higher yields in the presence of pollinators. In iris, a short‐tongued bumblebee shifted to more nectar robbing and longer flower handling time during legitimate flower visits than long‐tongued species because of increased competition with a florivorous sawfly (Ye et al., 2017). We did not find an effect of broad bean beetles on nectar robbing, but there was a negative effect on the proportion of legitimate flower visits. While nectar robbing is a common foraging behavior in the field (Marzinzig et al., 2018), further studies considering long‐tongued bumblebee species, which mainly conduct legitimate visits, are necessary to understand interactions between florivory and pollination in faba bean.

Differences in broad bean beetle effects at different levels (plant‐stand versus individual plant level) are likely due to differences between plants in the amount of florivory, oviposition, and pollination visits they received. At the plant‐stand level, taking into account variation in herbivory damage, bumblebee pollinators have a positive effect while broad bean beetles showed a tendency to interact with bumblebees and negatively affect individual bean weight. On the other hand, at the individual plant level, herbivory damage strongly and positively correlates with yield components but only in the absence of bumblebees. Effects of the broad bean beetle on yield components were only visible when high levels of pest damage per plant (>50%, see Figure 3a,b) were included in the analysis—damage levels, which are not occurring at plant‐stand level means in the cages, which are below 50% (Figure S3b). In the field, broad bean beetle damage is, to our knowledge, generally lower than 50% and so their potential to benefit yield might be less relevant for commercial production. In addition, the scenario of pollinators being absent, which is where the broad bean beetle benefitted yield components, is not realistic in the field. Therefore, under field conditions, it is unlikely that the broad bean beetle directly affects faba bean crop yield. In addition, these results should be generalized carefully, considering the high degree of nectar robbing performed by the pollinators and the potential that the broad bean beetle contributed to pollination in our study. However, the negative and indirect effects of the broad bean beetles on individual bean weight and proportion of legitimate visits by bumblebee pollinators call for further studies of these interactions in the field.

In summary, we confirm an insect pollination benefit on several faba bean yield components despite low rates of legitimate pollination, whereas plant responses to broad bean beetle herbivory differed at the individual versus plant‐stand level. Interestingly, positive effects of herbivory were found on faba bean yield components, but only in the absence of bumblebee pollinators. Another interesting result is that of a tendency for higher individual bean weight due to pollination, but only in the absence of broad bean beetles. Further studies at the plant level would be required to clarify how the plant allocates its resources under varying levels of pollination and herbivory. In addition, to disentangle the effects of pollen limitation from other factors, such as flower damage due to high visitation rates, it would be important to investigate effects of herbivory in hand pollinated plants. To our knowledge, this is the first experimental evidence of interactive effects of a lack of pollination and herbivory by broad bean beetle on faba bean plants. Our results strengthen the case for management of pollinators to maximize pollination benefits in faba bean. There was no evidence for direct yield losses (in terms of total bean weight and numbers) at infestation level of broad bean beetles that typically occur in the field despite high bean damage levels. Bean damage by the larvae will decrease faba bean salability for human consumption and germination and thus requires control. However, when damage thresholds are higher, as is the case when faba bean is cropped for animal fodder, our results indicate that there is less need to control for broad bean beetles, as there were no negative relationships between percentage of damaged beans and yield components. However, findings of a negative and indirect association between broad bean beetles and individual bean weight and proportion of legitimate visits by pollinators call for an improved understanding of these interactions in the field.

## AUTHOR CONTRIBUTION

**Laura Riggi:** Conceptualization (equal); Data curation (equal); Formal analysis (equal); Methodology (equal); Project administration (equal); Visualization (equal); Writing – original draft (equal); Writing – review & editing (equal). **Chloé Raderschall:** Conceptualization (equal); Methodology (equal); Project administration (equal); Supervision (equal); Writing – review & editing (equal). **Ola Lundin:** Conceptualization (equal); Formal analysis (supporting); Funding acquisition (lead); Investigation (equal); Methodology (equal); Project administration (equal); Resources (lead); Supervision (equal); Visualization (supporting); Writing – original draft (equal); Writing – review & editing (equal).

### OPEN RESEARCH BADGES

This article has earned an Open Data Badge for making publicly available the digitally‐shareable data necessary to reproduce the reported results. The data is available at [provided https://snd.gu.se/en].

## Supporting information

Supplementary Material

## Data Availability

Data will be made available via the free Swedish National Data Service (https://snd.gu.se/en).
