# Supplementary material for: Insect pest damage increases faba bean (Vicia faba) yield components but only in the absence of insect pollination
Source: Ecol Evol. 2022 Mar 7;12(3):e8686. doi: 10.1002/ece3.8686 (PMC8901888; doi:10.1002/ece3.8686)
Supplement: Supplementary file 1 — Supplementary Material [file ECE3-12-e8686-s001.docx]

**Supplementary material**


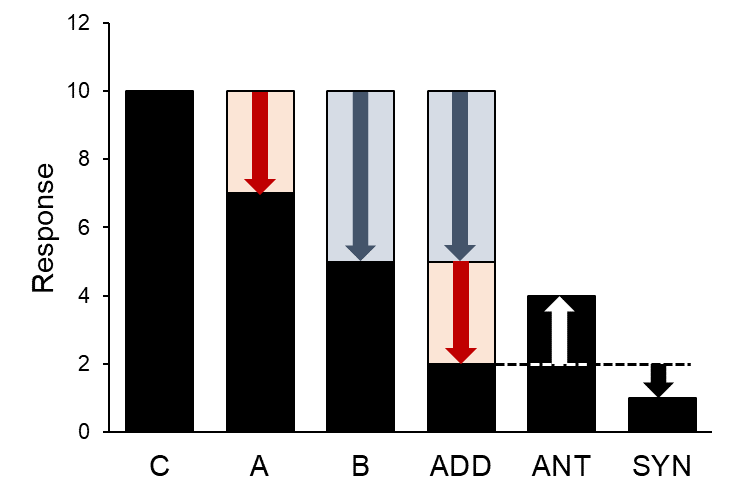


**Fig. S1.** **Conceptual figure of interaction types between two crop stressors on plant responses.** Treatments: non-stressed control (C), and hypothetical stressors A (e.g. herbivory) and B (e.g. lack of pollination). The effect of stressor A is the change in response due to A (C - A), in red. Similarly, the effect of stressor B is the change in response due to B (C - B), in blue. An interaction is additive (ADD) if response to the application of both stressor A and B is the sum of the effects of both treatments (response = (C - A) + (C - B)). The dashed line shows this additive prediction. The interaction of the stressors is antagonistic (ANT) if the change in the response is less than the sum of the effects of both stressors (response < (C - A) + (C – B)), and it is synergistic (SYN) if the change in the response is greater than the sum of the effects of both stressors (response > (C - A) + (C – B)) (modified from: Cote et al., 2016; Piggott et al., 2015) .

**
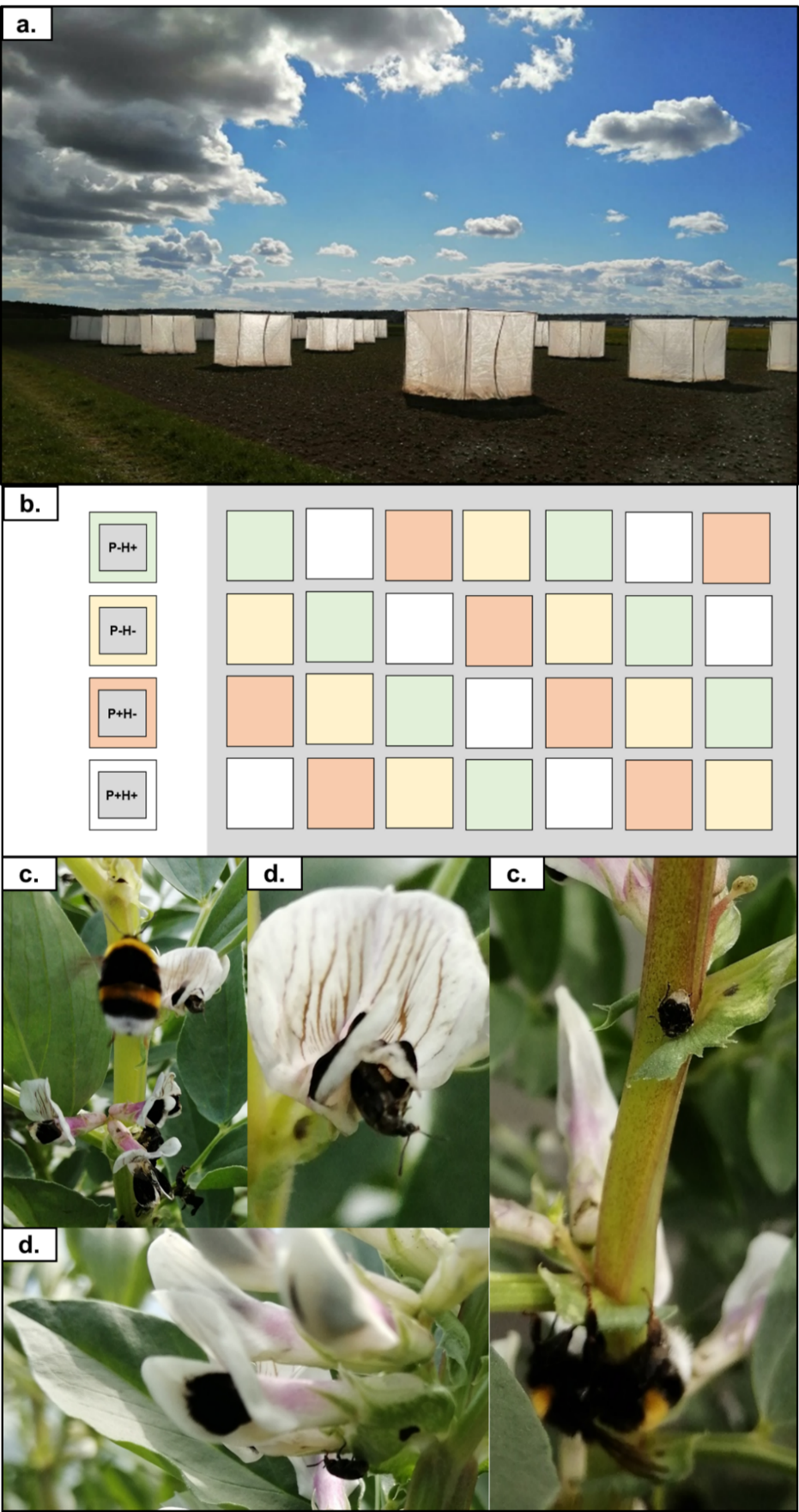
**

**Fig. S2.** Design of the experiment and pictures of the organisms involved. Figures (a.) shows the field set up with the 2 x 2 x 2 m cages built before bean emergence to avoid crop damage. The schematic representation in panel (b.) shows the arragements of the treatments in the field (Abbreviations: P = treatments with (+) and without (-) pollinators, H : treatments with (+) and without (-) *Bruchus rufimanus*). Photos showing *B. rufimanus* and *Bombus terrestris* near each other or on the same bean plant (c.) and *B. rufimanus* on the flowers (d.).


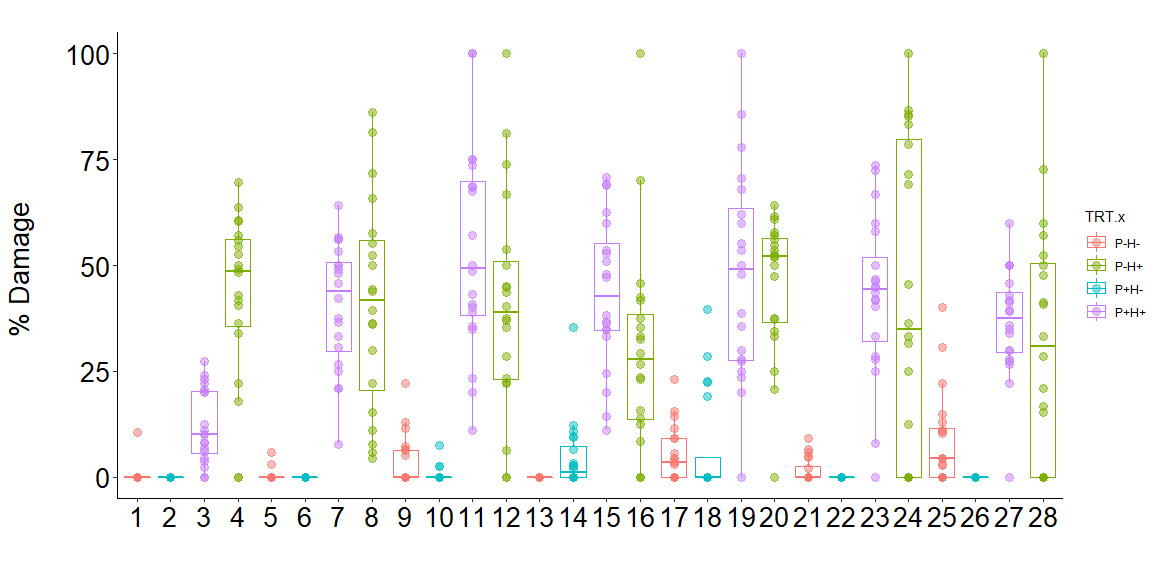


a.

b.


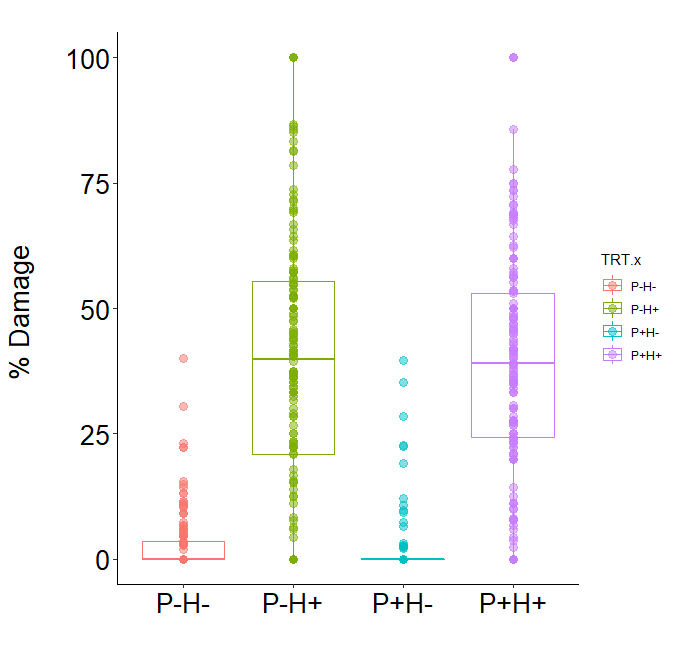


**Fig. S3.** Variation in percentage of *B. rufimanus* damage (measured as % of beans with emergence holes per plant) (a.) per cage and (b.) per treatment. Colors correspond to the four treatments combining pollinator (P-/P+) and herbivore (H-/H+) levels.

**
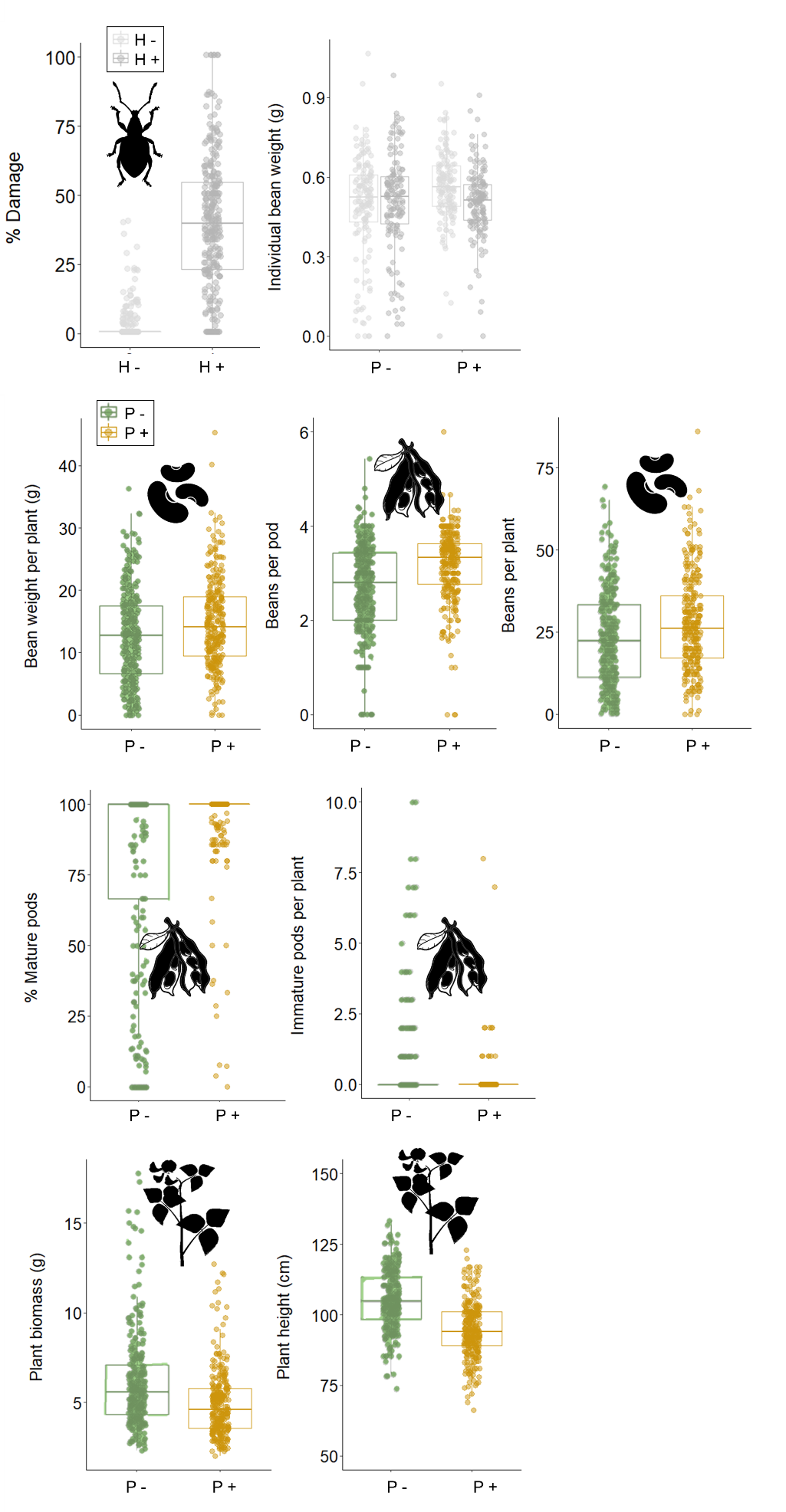
**

**Fig. S4. %** Damage, faba bean yield and growth components in relation to herbivory (H-/H+) and pollination (**P-/P+**) levels with raw data.

**
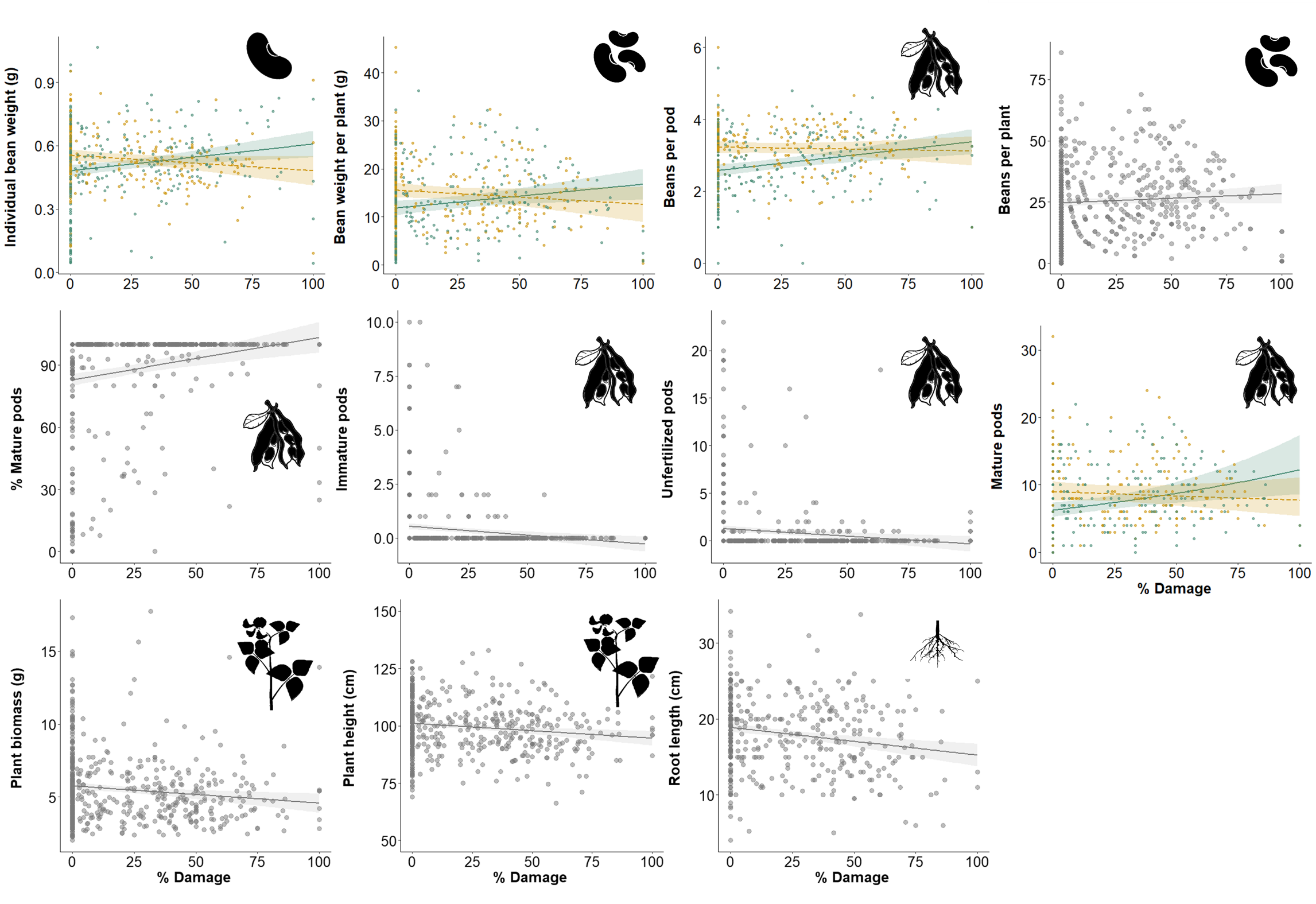
**

**Fig. S5.** Faba bean yield and growth components in relation to herbivory damage (% of beans with *B. rufimanus* emergence holes per plant) and pollination levels (**P-** : solid green line; and **P+**: dashed yellow line) with raw data. When interaction was not significant (see Table 1), overall data is presented (solid grey line). Bands represent 95% confidence intervals.

**Fig. S6**. Proportion of legitimate flower visits and robbing in relation to number open flowers per m^2^ before (black line) and after (green dotted line) sugar-water addition. Bands represent 95% confidence intervals.


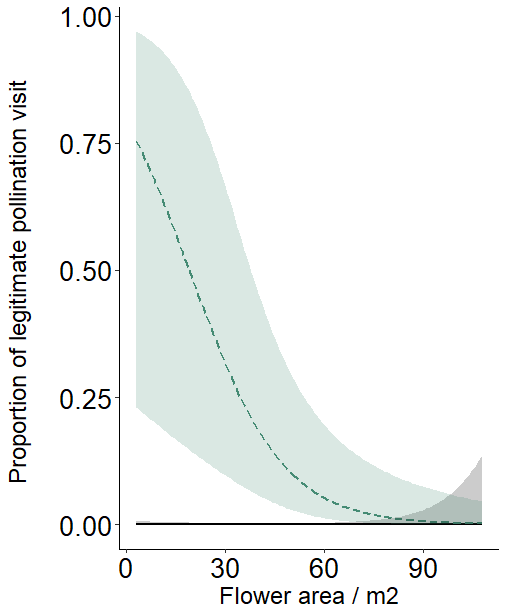

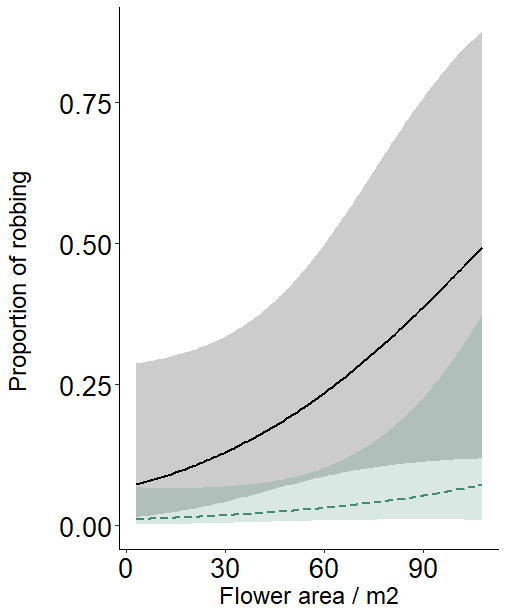


**Table S1.** Model outputs for plant-stand (cage) level linear mixed models after removal of cage 3. Only model results that differed significantly from the analyses including cage 3 are presented. Shown are mean estimates (Est) for the respective treatments, standard errors (se), p-values (p) and the adjusted marginal R^2^ (R_m_^2^) of the model. Significant results (p<0.05) are in bold.

| **Variables** |  | **H+** | **P+** | **H*P** | **R_m_^2^** |
| --- | --- | --- | --- | --- | --- |
| **Individual bean weight (g)** | **Est±se** | 0.01±0.02 | 0.06±0.02 | -0.07±0.03 | 0.03 |
|  | **p** | 0.59 | **0.005** | **0.037** |  |
| **Root length (cm)** | **Est±se** | -1.29±0.65 | 0.11±0.63 | 0.35±0.92 | 0.01 |
|  | **p** | **0.048** | 0.85 | 0.69 |  |
